# Assessment of medical management in Coronary Type 2 Diabetic patients with previous percutaneous coronary intervention in Spain: A retrospective analysis of electronic health records using Natural Language Processing

**DOI:** 10.1371/journal.pone.0263277

**Published:** 2022-02-10

**Authors:** Carlos González-Juanatey, Manuel Anguita-Sá́nchez, Vivencio Barrios, Iván Núñez-Gil, Juan Josá Gómez-Doblas, Xavier García-Moll, Carlos Lafuente-Gormaz, María Jesús Rollán-Gómez, Vicente Peral-Disdie, Luis Martínez-Dolz, Miguel Rodríguez-Santamarta, Xavier Viñolas-Prat, Toni Soriano-Colomé, Roberto Muñoz-Aguilera, Ignacio Plaza, Alejandro Curcio-Ruigómez, Ernesto Orts-Soler, Javier Segovia, Claudia Maté, SAVANA Research Group, Ángel Cequier

**Affiliations:** 1 Hospital Universitario Lucus Augusti, Lugo, Spain; 2 Hospital Universitario Reina Sofía, Córdoba, Spain; 3 Hospital Universitario Ramón y Cajal, Madrid, Spain; 4 Hospital Clínico Universitario San Carlos, Madrid, Spain; 5 Hospital Universitario Virgen de la Victoria, Málaga, Spain; 6 Hospital Universitario Santa Creu i Sant Pau, Barcelona, Spain; 7 Hospital Universitario de Albacete, Albacete, Spain; 8 Hospital Universitario Río Hortega, Valladolid, Spain; 9 Hospital Universitario Son Espases, Palma de Mallorca, Spain; 10 Hospital Universitario La Fe, Valencia, Spain; 11 Hospital Universitario de León, León, Spain; 12 Hospital Vall d’Hebron, CIBERCV, Barcelona, Spain; 13 Hospital Infanta Leonor, Madrid, Spain; 14 Hospital Infanta Sofía, Madrid, Spain; 15 Hospital Universitario de Fuenlabrada, Madrid, Spain; 16 Hospital General Universitario de Castellón, Castellón, Spain; 17 Hospital Universitario Puerta de Hierro, Madrid, Spain; 18 Savana, Madrid, Spain; 19 Hospital Universitario de Bellvitge and Universidad de Barcelona, IDIBELL, Barcelona, Spain; University of Bologna, ITALY

## Abstract

**Introduction and objectives:**

Patients with type 2 diabetes (T2D) and stable coronary artery disease (CAD) previously revascularized with percutaneous coronary intervention (PCI) are at high risk of recurrent ischemic events. We aimed to provide real-world insights into the clinical characteristics and management of this clinical population, excluding patients with a history of myocardial infarction (MI) or stroke, using Natural Language Processing (NLP) technology.

**Methods:**

This is a multicenter, retrospective study based on the secondary use of 2014–2018 real-world data captured in the Electronic Health Records (EHRs) of 1,579 patients (0.72% of the T2D population analyzed; n = 217,632 patients) from 12 representative hospitals in Spain. To access the unstructured clinical information in EHRs, we used the *EHRead*^*®*^ technology, based on NLP and machine learning. Major adverse cardiovascular events (MACE) were considered: MI, ischemic stroke, urgent coronary revascularization, and hospitalization due to unstable angina. The association between MACE rates and the variables included in this study was evaluated following univariate and multivariate approaches.

**Results:**

Most patients were male (72.13%), with a mean age of 70.5±10 years. Regarding T2D, most patients were non-insulin-dependent T2D (61.75%) with high prevalence of comorbidities. The median (Q1-Q3) duration of follow-up was 1.2 (0.3–4.5) years. Overall, 35.66% of patients suffered from at least one MACE during follow up. Using a Cox Proportional Hazards regression model analysis, several independent factors were associated with MACE during follow up: CAD duration (p < 0.001), COPD/Asthma (p = 0.021), heart valve disease (p = 0.031), multivessel disease (p = 0.005), insulin treatment (p < 0.001), statins treatment (p < 0.001), and clopidogrel treatment (p = 0.039).

**Conclusions:**

Our results showed high rates of MACE in a large real-world series of PCI-revascularized patients with T2D and CAD with no history of MI or stroke. These data represent a potential opportunity to improve the clinical management of these patients.

## Introduction

Type 2 diabetes (T2D) has reached epidemic proportions globally due to a steady increase in life expectancy, high prevalence of obesity and sedentary lifestyle, and pervasive unhealthy eating habits [1]. In 2019, the global prevalence of diabetes was estimated to be around 9%. By 2030, the disease is expected to reach 700 million people [2].

In T2D patients, the progressive atherosclerotic disease leads to a twofold increased risk for cardiovascular diseases (CVD), including myocardial infarction (MI), stroke, peripheral vascular disease, and coronary artery disease (CAD) [3–5]. In addition, admission hyperglicemia is a strong predictor of short- and long-term adverse outcomes in patients with acute MI [6]. Notably, CAD is the main cause of mortality in patients with T2D, and diabetes leads to a 2- to 4-fold increased risk of death due to heart disease [7, 8]. Indeed, approximately one third of patients undergoing percutaneous coronary intervention (PCI) are diabetic [8]. PCI procedures, particularly with drug-eluting stents, have proven successful in the management of stable angina and improving quality of life in patients with diabetes and CAD [9].

Patients with T2D and stable CAD who have been revascularized with PCI are at high risk of ischemic events. In these patients, international guidelines recommend the use of antiplatelet therapy to improve cardiovascular outcomes following intervention [10–12]. However, evidence supporting the long-term use of dual antiplatelet regimens in patients with T2D and CAD but without a history of MI or stroke has been inconclusive. In this context, the THEMIS-PCI trial was part of a recent large phase 3 randomized, double-blinded, placebo-controlled trial designed to evaluate the efficacy and safety of ticagrelor 60 mg bid added to background acetylsalicylic acid (ASA) therapy for the prevention of MACE in this population [13]. This trial showed that the incidence of ischemic cardiovascular events over a 3.3-year follow-up was significantly lower in the ticagrelor group (7.7%) than in the placebo group (8.6%). On the other hand, in a recent population-based cohort study aimed at comparing the risk of MACE with ticagrelor vs clopidogrel in patients with acute coronary syndrome (ACS) and previous PCI intervention, ticagrelor was not associated with a reduction in MACE in the year following revascularization. as compared with clopidogrel [14].

In summary, the available evidence indicates that a) T2D is an important risk factor for CAD and that diabetic status may worsen clinical outcomes after PCI and other revascularization procedures [15–18], b) revascularized patients are at high risk of ischemic events, and c) further research regarding treatment outcomes regarding MACE in these patients is warranted. Thus, a thorough and updated clinical characterization of these patients in real-world settings becomes critical to design early intervention strategies, improve prognosis, and ultimately reduce cardiovascular events.

The present study aimed to provide real-world insights into the clinical characteristics and management of patients with CAD, T2D, and a previous history of PCI (but no prior MI or stroke) in Spain by analyzing readily available information in the Electronic Health Records (EHRs) of the Spanish National Healthcare System.

## Materials and methods

The present study was classified as a ‘non-post-authorization study’ by the Spanish Agency of Medicines and Health Products (AEMPS) and was approved by the Institutional Review Board of each participating hospital. This study was conducted in compliance with legal and regulatory requirements and followed generally accepted research practices described in the Helsinki Declaration in its latest edition, Good Pharmacoepidemiology Practices, and applicable local regulations. Patient consent was not required in this study since data were retrospectively captured from patients’ EHRs in an anonymized, and aggregated in an irreversible dissociated manner.

### Data source and study design

**Fig 1:**
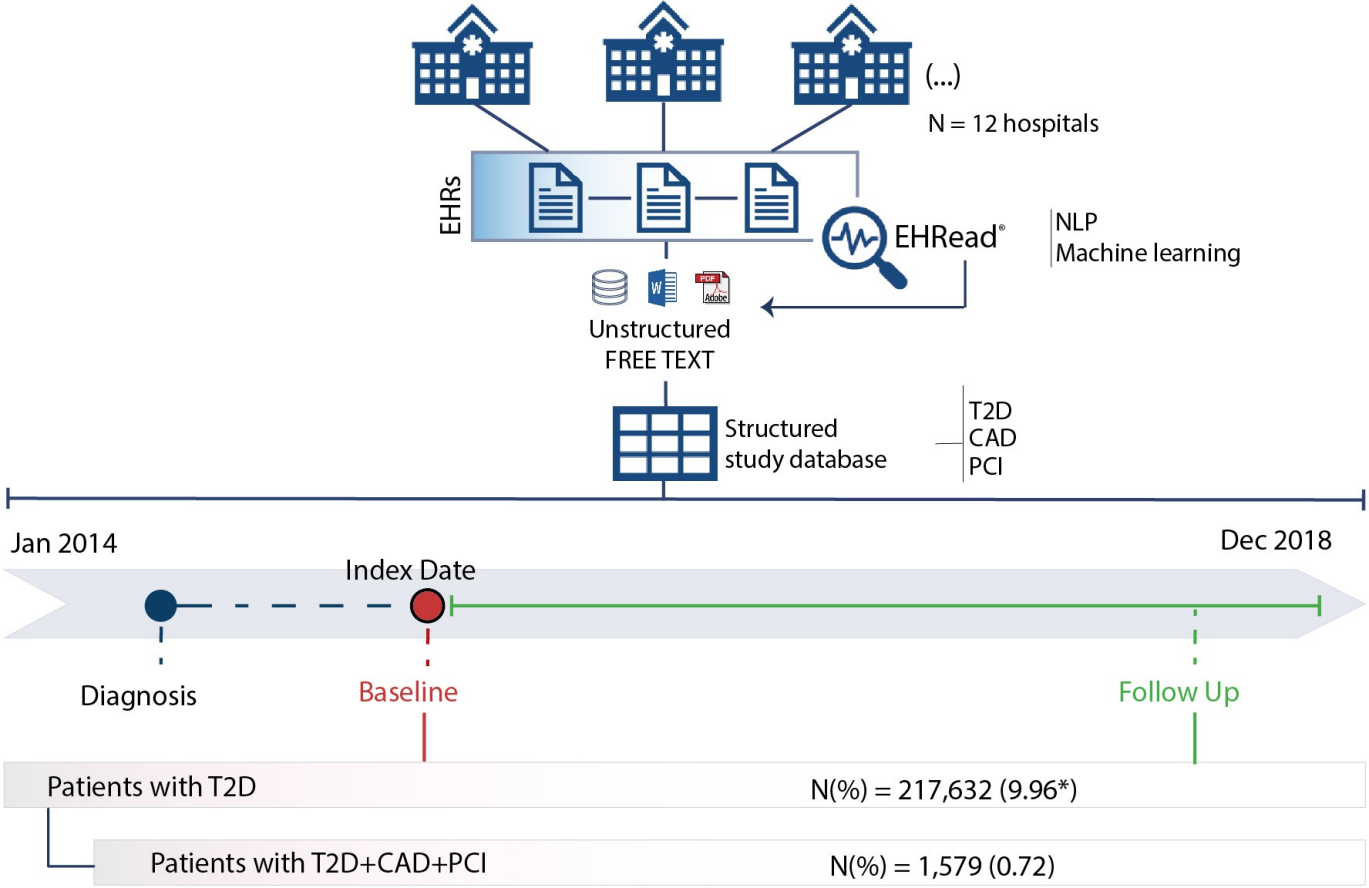
Study design and timeline. The Index Date (i.e., Baseline) was defined as the timepoint when diagnostic criteria for both T2D and CAD were first identified in patients who underwent PCI. All available EHRs prior to January 2014 were considered to extract information regarding the clinical history of patients (dotted blue line). The follow-up period ranged from the Index Date to the end of the study period or the last data point available. Unstructured data from patients’ EHRs was extracted and organized with the EHRead^®^ technology. See the Methods section for further details. *Estimated prevalence data for T2D calculated over the total patient population at midpoint of the study period minus both patients who died in the hospital during the study period (N = 41,747) and patients without follow-up data in the 12 months prior to midpoint (N = 1,460,161).

This was a real-world, multicenter, and retrospective study based on the secondary use of the unstructured data captured in the EHRs of 12 representative hospitals from 6 major regions (namely Madrid, Catalonia, Valencia, Balearic Islands, Castilla-La Mancha, and Castilla León) within the Spanish National Healthcare System (Fig 1). Data were collected between January 1, 2014 and December 31, 2018 from all available services and departments in each participating site (including inpatient hospital, outpatient hospital, and emergency room).

A cross-sectional analysis of the study variables (including demographic and clinical characteristics, comorbidities, medical therapy, and treatment) was performed at Index Date (Fig 1), defined as the timepoint when mentions of both T2D and CAD are first found in EHRs. The rates and incidence of MACE (MI, ischemic stroke, urgent coronary revascularization, and hospitalization due to unstable angina) was analyzed during follow up, which comprised the time between the baseline and end of the study period (Fig 1).

### Study population and eligibility criteria

#### Inclusion criteria

To be eligible for inclusion in the study, patients had to fulfil all of the following inclusion criteria:

- ≥18 years old
- Diagnosis of both T2D and CAD
- Revascularization with PCI
- Documented ongoing use of glucose-lowering drugs (oral hypoglycemic agents) for at least 6 months
- Available follow-up information spanning at least 6 months

#### Exclusion criteria

Patients were excluded from the study if they met any of the following criteria:

- Prior MI
- Prior stroke (except Transient Ischemic Attack)
- Prior intracranial bleeding
- Gastrointestinal bleeding within the last 6 months
- Renal failure requiring dialysis
- History of liver cirrhosis or liver cancer

### Extracting the unstructured free text from EHRs

To access the unstructured clinical information in EHRs, we used Savana’s *EHRead*^*®*^ technology [19–24]. Based on NLP and machine learning, this technology facilitates the extraction of information from patients’ EHRs and subsequent standardization of extracted clinical concepts to a common terminology. The clinical corpora used by *EHRead* is based on SNOMED Clinical Terminology and includes more than 300,000 medical concepts, acronyms, and laboratory parameters. These concepts are later organized based on EHR sections (medical history, laboratory results, prescriptions, procedures, diagnoses, etc.), hospital service, and other specifications.

*EHRead*’s ability to correctly identify patient records containing key variables associated with the study disease were assessed according to previously published procedures [21], summarized in S1 File. Briefly, the evaluation of *EHRead*’s performance consists of a comparison between *EHRead’*s reading output and an annotated corpus of EHRs by expert physicians (‘gold standard’). The result of this comparison is expressed in terms of the standard metrics of accuracy (P), recall (R), and their harmonic mean F1-score. As shown in S1 Table in S1 File, our evaluation yielded a F1-Score of ≥90% in most analyzed variables, showing a near-optimal performance in *EHRead*’s ability to properly identify most records that contain T2D, CAD, and related variables.

### Data analyses

Frequency and summary tables were used to display information for categorical and continuous variables, respectively. The association between MACE rates and the variables included in this study was evaluated following two different approaches. In the univariate approach, a model was fitted to the study population for each variable at baseline. The association between MACE and categorical variables was assessed with Fisher’s exact tests. Independent samples two-tailed T-tests were performed to assess statistical differences between patients with and without MACE for each continuous numeric variable. Welch’s adjustment was incorporated for unequal variances. Mann-Whitney U tests were performed instead if the normality assumption (Shapiro-Wilk test) was not met. Significant differences were considered when p < 0.05 in two-tailed tests. In the multivariate-survival approach, a data-driven variable and model selection was performed. First, variables with missing values (all laboratory tests had >20% missing values) or with zero variance (CAD and stroke) were excluded. Then, multicollinearity was assessed and variables with a high variance inflation factor (VIF) were excluded (atrial fibrillation; VIF > 5). The remaining variables (VIF < 2) were used to fit a Cox proportional hazards (PH) survival model in the study population. Then, using Akaike information criterion (AIC), variable selection and model evaluation were performed in a stepwise manner until reaching a model with the optimal explanatory variables. Significant differences were considered when p < 0.05 in two-tailed tests.

## Results

EHRs from 2,185,060 patients were processed from 12 participating hospitals between January 1, 2014 and December 31, 2018. The estimated prevalence of T2D in the hospital population was 9.96% (n = 217,632). The target population (patients diagnosed with T2D, CAD, and documented PCI revascularization with no previous history of MI or stroke) comprised a total of 1,579 patients (0.72% of the T2D population; Fig 1). The demographic and clinical characteristics of patients at time of inclusion in the study are shown in Table 1. Most patients were male (72.13%; n = 1,139), with a mean age of 70.5±10 years.

**Table 1:** Demographics, substance use, vital signs, and comorbidities at baseline.

	N (%)
	1,579(100)
*Demographics*	
Gender	
Female	435(27.55)
Male	1139(72.13)
Age (years)	
Mean(SD)	70.5(10)
Median	71
(Q1-Q3)	(64–78)
Missing	5
*Substance use* *	
Tobacco	
Ex/Former smoker	765(48.45)
No/Unknown	617(39.08)
Yes	197(12.48)
*Vital signs*	
Heart rate (bpm)	
N	1011
Mean(SD)	64.4(22.8)
Median	68
(Q1-Q3)	(58–78)
Missing*	568
Systolic blood pressure (mmHg)	
N	951
Mean(SD)	141.5(22.2)
Median	140
(Q1-Q3)	(127–156)
Missing*	628
Diastolic blood pressure (mmHg)	
N	951
Mean(SD)	74.3(13.1)
Median	73
(Q1-Q3)	(66–82)
Missing*	628
*Comorbidities* **	
*Blood and lymphatic system disorders*	
Anemia	290(18.37)
*Cardiovascular disorders*	
Coronary Heart Disease (CHD)	1579(100)
Transient Ischemic Attack (TIA)	44(2.79)
Arterial hypertension	1396(88.41)
Moderate/severe LV systolic dysfunction	127(8.04)
Heart Failure	361(22.86)
Atrial Flutter	270(17.1)
Atrial fibrillation	212(13.43)
Heart Valve Disease	659(41.74)
Peripheral Vascular Disease (PVD)	621(39.33)
Other/Unknown	595(37.68)
Peripheral Artery Disease (PAD)	276(17.48)
Claudication	137(8.68)
Foot or leg cellulitis-osteomyelitis	16(1.01)
Other/Unknown	216(13.68)
Angina	1083(68.59)
Unstable angina	564(35.72)
Stable angina	519(32.87)
Other/Unknown	730(46.23)
*Eye disorders*	
Diabetic retinopathy	118(7.47)
*Endocrine*, *metabolism*, *and nutrition disorders*	
Hyperlipidemia	638(40.41)
Hypoglycemia	69(4.37)
Gout	65(4.12)
Hyperthyroidism	27(1.71)
Hypothyroidism	104(6.59)
*Gastrointestinal and hepatobiliary disorders*	
Chronic liver disease	29(1.84)
*Musculoskeletal and connective tissue disorders*	
Diabetic foot	9(0.57)
*Nervous system disorders*	
Diabetic neuropathy	41(2.60)
*Psychiatric disorders*	
Depression/Anxiety	249(15.77)
*Renal and urinary disorders*	
CKD (Chronic Kidney Disease)	235(14.88)
*Reproductive system and breast disorders*	
Erectile dysfunction	35(2.22)
*Respiratory*, *thoracic and mediastinal disorders*	
COPD/Asthma	269(17.04)
Sleep apnea	203(12.86)

* Missing data resulting from extracting laboratory results from unstructured information captured in the EHRs.

** Data indicate single diagnostic labels (i.e., if diagnostic information for a given condition exists multiple times for a single patient, the comorbid condition was counted only once). In addition, a single patient could have been diagnosed with more than one of the analyzed medical conditions.

Cardiovascular diseases (other than CAD) and endocrine/metabolic disorders were the most common comorbidities in the target population (Table 1); 88.41% (n = 1,369) of patients suffered from hypertension, 68.59% (n = 1,083) angina, 41.74% (n = 659) valvular disease, 30.53% (n = 482) atrial fibrillation/atrial flutter, and 22.86% (n = 361) heart failure. A diagnosis of hyperlipidemia was found in 40.41% (n = 638) of the patients and tobacco use in 25% (n = 394). Regarding respiratory disorders, COPD/Asthma was present in 17.04% of patients (n = 269) and sleep apnea in 12.86% (n = 203). Chronic kidney disease was diagnosed in 17.04% (n = 269) of the patients, peripheral artery disease in 17.48% (n = 276), diabetic retinopathy in 7.47% (n = 118), and diabetic neuropathy in 2.6% (n = 41).

As shown in Table 2, most patients were non-insulin-dependent T2D (61.75%; n = 975). Regarding CAD type, more than half of patients had been diagnosed with MVD (58.52%; n = 924), a third of the population had single-vessel CVD (33.69%; n = 532,) and 1.37% (n = 20) of patients had a diagnosis of left main artery coronary disease (LMACD). Table 2 also shows the time passed since PCI, CABG, and coronary angiography were last performed at time of analysis.

**Table 2:** T2D- and CAD-related clinical characteristics.

	N (%)
	1,579(100)
*T2D*	
Type of T2D	
Insulin-dependent	604(38.25)
Non-insulin-dependent	975(61.75)
Age at diagnosis	
Mean(SD)	4.6 (6.6)
Median (Q1-Q3)	67 (58–75)
Missing	8
*CAD*	
Type of CAD	
Single coronary vessel disease	532(33.69)
Multivessel coronary disease	924(58.52)
Left main coronary artery disease	20(1.27)
Other	103(6.52)
Age at diagnosis	
Mean(SD)	67.3 (10.7)
Median (Q1-Q3)	68 (59–75)
Missing	5
Procedures	
PCI*	1579(100)
CABG*	218(13.81)
Coronary angiography	1387(87.84)
Time since most recent PCI (years)	
Mean(SD)	3.2 (4.6)
Median (Q1-Q3)	1.2 (0.4–4.2)
Missing	519
Time since most recent CABG (years)	
Mean(SD)	6.6 (7.1)
Median (Q1-Q3)	3.6 (1.2–10.2)
Missing	56

* Calculated over patients who underwent revascularization. PCI = Percutaneous Coronary Intervention; CABG = Coronary Artery Bypass Graft.

We also used the NLP system to extract unstructured information on laboratory results captured by physicians in their clinical notes. Despite the relatively high proportion of patients with missing unstructured laboratory information in their EHRs, we obtained data for several laboratory parameters for more than half of the study sample (S2 Table in S1 File). At inclusion in the study, the median (Q1-Q3) HbA1c was 7.1% (6.4–8), HDL was 40 mg/dl (33–47), and LDL 77 mg/dl (62–96.6).

**Fig 2:**
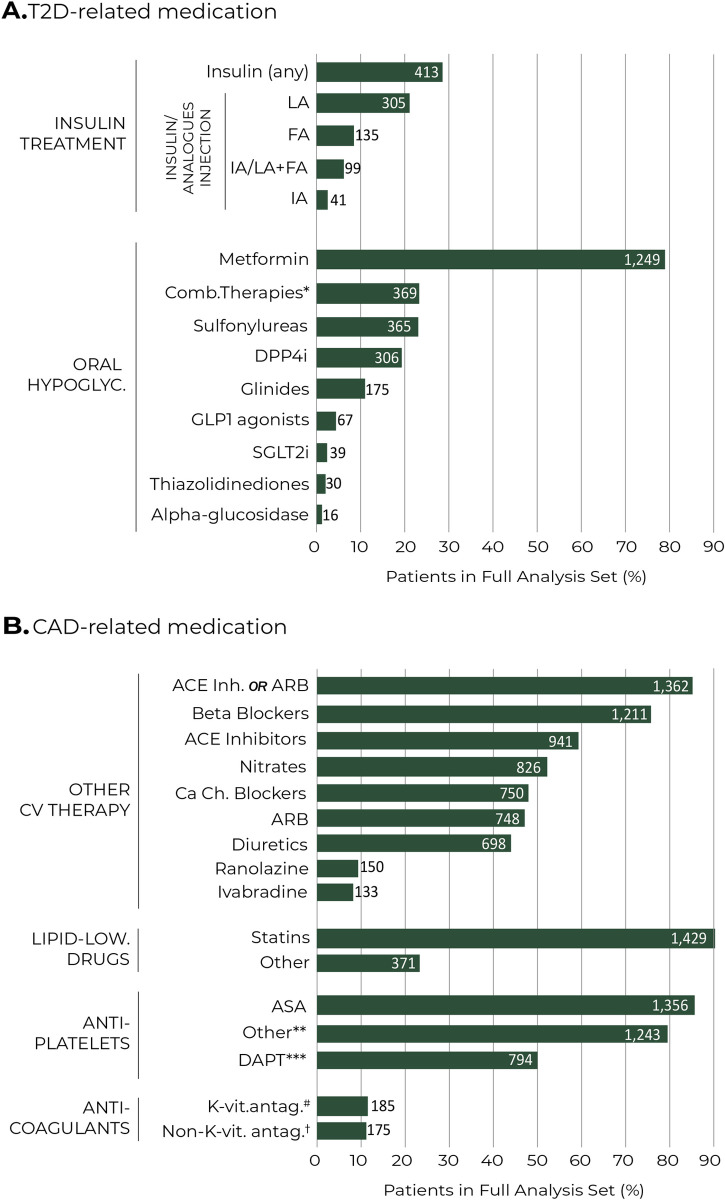
T2D- and CAD-related medication at baseline. Percentage of patients prescribed with different medications for T2D (A) and CAD (B). Numbers within bars represent number of patients. *Any fixed combination of two of the above oral hypoglycemic agents (i.e., two or more active substances combined in one single prescription). **Other include clopidogrel, prasugrel, ticagrelor, and other lipid-lowering drugs. ***DAPT refers to ASA plus other anti-platelet drug. ^#^K-vitamin antagonists include warfarin (n = 2; 0.13%) and acenocumarol (n = 185; 11.72%) ^†^Non-K-vitamin antagonists include heparins (n = 121; 7.66%), direct thrombin inhibitors (n = 34; 2.15%), direct factor Xa inhibitors (n = 23; 1.46%), and fondaparinux (n = 14; 0.89%). ACE = angiotensin-converting enzyme inhibitors; ARB = angiotensin II receptor blockers; ASA = acetylsalicylic acid; DAPT = dual antiplatelet therapy; DPP4i = dipeptidyl peptidase 4 inhibitors; SGLT2i = sodium-glucose cotransporter 2 inhibitors; GLP1 = Glucagon-like peptide-1; FA = Fast acting; IA = Intermediate acting; LA = Long acting.

The pharmacological treatments prescribed for the management of T2D and CAD are show in Fig 2A and 2B, respectively. As for oral hypoglycemic agents, the most used single-drug treatments were metformin (79.1%; n = 1,249), sulfonylureas (23.12%; n = 365), and DPP4i (19.38%; n = 306). Insulin treatment was documented in 26.16% (n = 413) of patients (Fig 2A). As depicted in Fig 2B, we gathered information regarding cardiovascular treatments: statins (90.5%; n = 1,429), ACE inhibitors or ARBs (86.26%; n = 1,362) and beta blockers (76.69%; n = 1,211). As for oral antiplatelet agents, the most prescribed were ASA (85.88%; n = 1,356) and dual antiplatelet therapy (50.28%; n = 794).

**Fig 3:**
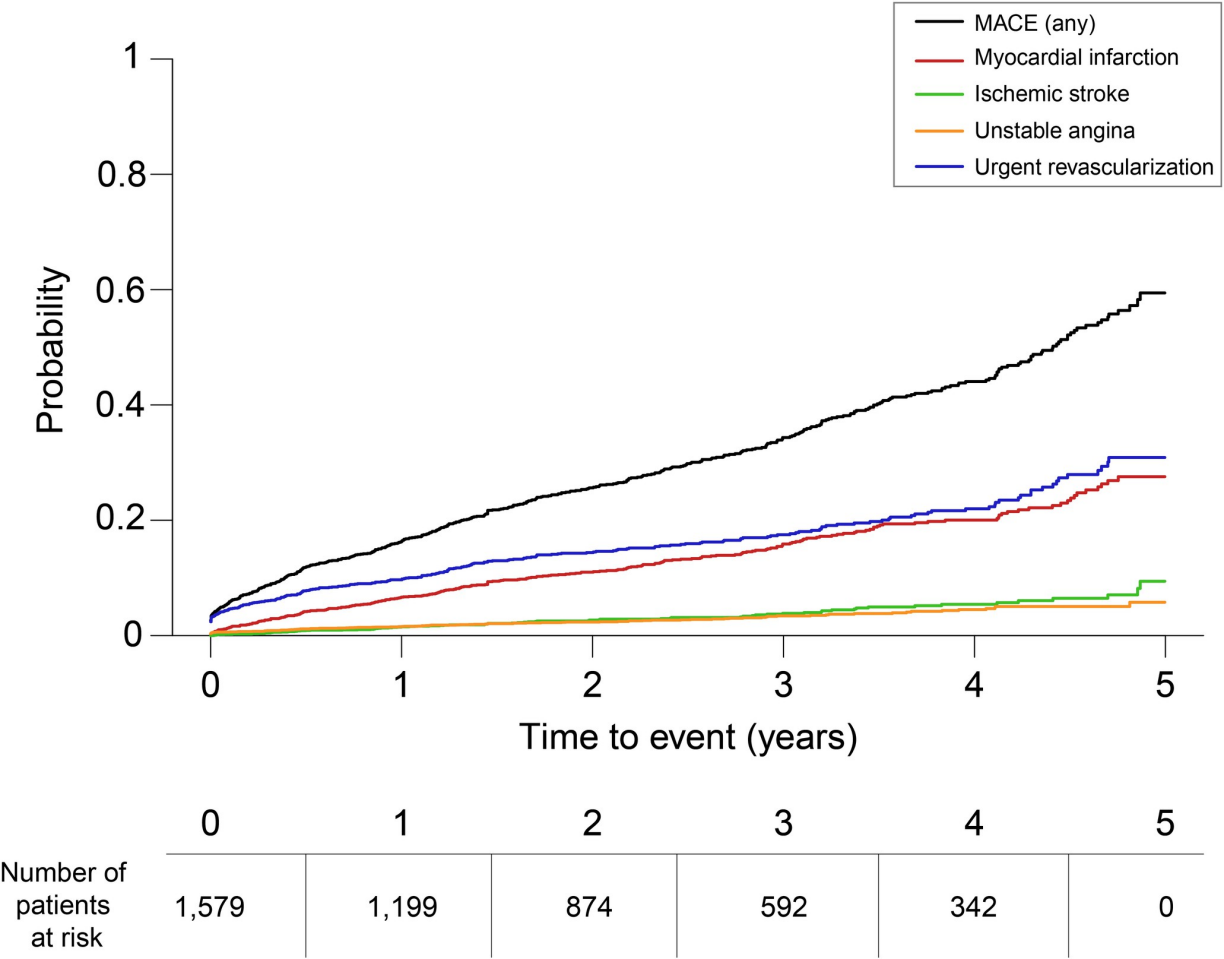
Probability of MACE over time during the follow-up period. Probability for any MACE event (black), myocardial infarction (red), ischemic stroke (green), hospitalization for unstable angina (orange), and urgent revascularization (blue) during follow up. The number of patients at risk (same for all categories) across the follow-up period is indicated below.

The median (Q1, Q3) follow-up duration was 1.2 (0.3–4.5) years. During this period, this study aimed to document the cumulative incidence and rates of MACE (MI, ischemic stroke, hospitalization for unstable angina, and urgent coronary revascularizations). Because MACE were aligned to the nature of the data source and methodology used, all-cause or CV death cannot be included in the analysis (EHRs only capture in-hospital death). S3 Table in S1 File shows the overall incidence rates and cumulative incidence of MACE; 35.66% (n = 563) of patients suffered from at least one MACE event during follow up. The probability of suffering any MACE as well as MACE subtypes over the follow-up period is shown in Fig 3.

Finally, we sought to determine the clinical characteristics associated with MACE during follow up. In a univariate analysis, T2D and CAD disease durations (both p = 0.001), heart failure (p = 0.004), MVD (p = 0.005), diabetic retinopathy (p = 0.012), and COPD/asthma (p = 0.014) showed statistically significant association with MACE in the follow-up period (S4 Table in S1 File). Regarding treatments, MACE was associated with prescription of insulin (p < 0.001), antiplatelet agents (p = 0.001), diuretics (p = 0.006), and statins (p = 0.002; S4 Table in S1 File).

Using a Cox Proportional Hazards regression model analysis, we found 7 independent predictors of MACE occurrence during follow up, including CAD duration (HR = 0.77; 95% CI = 0.72–0.84; p < 0.001), COPD/Asthma (HR 1.28; 95% CI = 1.04–1.59; p = 0.021), heart valve disease (HR = 1.22; CI 95% = 1.02–1.46; p = 0.031), MVD (HR = 1.27; 95% CI = 1.07–1.51; p = 0.005), insulin treatment (HR = 1.53; CI 95% = 1.26–1.85; p < 0.001), statins treatment (HR = 0.62; CI 95% = 0.48–0.81; p < 0.001), and clopidogrel treatment (HR = 0.83; CI 95% = 0.70–0.99; p = 0.039) (Table 3).

**Table 3:** Multivariate model of factors associated with the occurrence of MACE during follow up.

	HR* (CI 95%*)	*P* value**
CAD: Time since first mention in EHRs	0.77 (0.72, 0.84)	< 0.001**
TIA	1.51 (0.96, 2.38)	0.073
Heart failure	1.23 (1.00, 1.52)	0.053
Heart valve disease	1.22 (1.02, 1.46)	0.031**
Multivessel coronary disease	1.27 (1.07, 1.51)	0.005**
Diabetic retinopathy	1.28 (0.95, 1.73)	0.102
Diabetic neuropathy	0.63 (0.37, 1.10)	0.107
CKD	0.82 (0.64, 1.05)	0.114
COPD/Asthma	1.28 (1.04, 1.59)	0.021**
*Treatments*		
Sulfonylureas	1.18 (0.98, 1.44)	0.086
Alpha-glucosidase	0.30 (0.07, 1.19)	0.087
Insulin	1.53 (1.26, 1.85)	< 0.001**
Vitamin-k antagonist oral anticoagulant	0.77 (0.59, 1.02)	0.073
Clopidogrel	0.83 (0.70, 0.99)	0.039**
Ranolazines	1.30 (0.99, 1.70)	0.058
Statins	0.62 (0.48, 0.81)	< 0.001**

**Statistical differences between MACE and No-MACE patients were considered when p < 0.05 in two-tailed tests. HR = Hazards ratio; CI = Confidence Interval; CAD = Coronary artery disease; TIA = Transient ischemic attack; CKD = Chronic kidney disease; COPD = Chronic obstructive pulmonary disease.

## Discussion

Using NLP and machine learning techniques, we were able to access and analyze the free-text clinical information in the EHRs of a large series of patients with T2D and CAD with no history of MI or stroke who underwent PCI revascularization in Spain. Our results provide a thorough characterization of these patients, including demographics, disease characteristics, comorbidities, medical management, and clinical factors associated with the occurrence of MACE.

Our study sample was extracted from a target population of 217,632 T2D patients. This number represents an estimated prevalence of diagnosed T2D of 9.9%. This estimate, calculated in the hospital population, is slightly higher than those previously reported in the general Spanish population yet falls within the 7%-14% age-adjusted range in the last ten years using classic epidemiological methods [25–30].

The patients in our series were predominantly male, older than 70 years, and almost 60% of them suffered from MVD. Most patients were being treated with cardiovascular prevention medication (statins: 90.5% of patients, ACEi/ARBs: 86.2%, beta blockers: 76.6%, and antiplatelet therapy: 85.8%), while 50.2% were treated with dual antiplatelet therapy. These demographics and treatment data are aligned with the recent observational study using 2013–2014 data from the Diabetes Collaborative Registry linked to Medicare administrative claims (ATHENA study) [31]. In this cohort, the distribution of cardiovascular prevention medication was as follows: statins 84.2%, ACEi/ARBs 80%, beta blockers 79.2%, at least one antiplatelet agent 91.3%, and dual antiplatelet therapy in 32% of the patients. In addition, a drug-eluting stent was implanted in more than half of the patients (thus comparable to the 60% in the THEMIS-PCI trial) and the time since the most recent PCI was 3.2 years, again very similar to the THEMIS-PCI trial patients [13]. These findings indicate that the PCI population represents a very-high-risk patient group among those with CAD and concomitant T2D and suggest similar levels of care documented in both clinical trials and our real-world study.

The reported treatment data must be interpreted considering existing clinical recommendations. Current clinical guidelines recommend the use of antiplatelet therapies in patients with T2D and prior CV disease, but not in those with low CV risk [11]. However, guidelines are less clear on their recommendations for the use of antiplatelet therapies in patients with T2D and established CV disease without previous ischemic events. Recent clinical trials have addressed whether antiplatelet therapies reduce the incidence of ischemic events in patients with T2D. The ASCEND trial (A Study of Cardiovascular Events iN Diabetes) assessed the absolute benefits of ASA in patients with T2D and no evident CV disease and showed that the prevention of serious vascular events was largely counterbalanced by bleeding risk [32]. On the other hand, dual antiplatelet regimens demonstrated a clear benefit in patients with T2D and a previous history of MI exceeding 1 year. In the PEGASUS-TIMI 54 trial (Prevention of Cardiovascular Events in Patients With Prior Heart Attack Using Ticagrelor Compared to Placebo on a Background of Aspirin-Thrombolysis In Myocardial Infarction 54), patients with T2D had a greater absolute reduction in the risk of major cardiovascular events (cardiovascular death, MI or stroke) than patients without T2D when treated with a combination of ticagrelor and ASA [33]. Finally, the THEMIS trial, was designed to evaluate the efficacy and safety of ticagrelor 60mg bid added to ASA therapy for the prevention of major CV events in patients with T2D and established CAD and without a history of previous MI or stroke [34]. This study showed that the incidence of ischemic events was lower in patients receiving ASA and ticagrelor than in those receiving ASA and placebo. However, the incidence of major bleeding events was significantly higher in the ticagrelor group than in the placebo group [34]; in spite of these results, a significantly net clinical benefit was demonstrated in the pre-specified subgroup analysis of patients who underwent PCI (THEMIS-PCI) [13]. Thus, the long-term dual antiplatelet therapy could be beneficial in T2D patients with stable CAD and previous PCI, with low bleeding risk and high ischemic risk.

Available evidence indicates that T2D leads to impaired microvascular function [35] and increased risk for MACE in patients with stable manifestations of atherothrombosis. In addition, although adjunctive procedures such as thrombus aspiration prior to PCI intervention may improve ST-elevation MI (STEMI) outcomes in hyperglycemic patients [36], revascularized patients with T2D and stable CAD are still at high risk of ischemic events. Indeed, hyperglycemic-STEMI patients can experience adverse cardiovascular events such as restenosis and no-reflow despite PCI intervention [37, 38]. Notably, a recent study pointed to the involvement of the miR33/SIRT1 protein pathway in the inflammatory and coagulative processes of hyperglycemic coronary thrombi, which was in turn associated with rehospitalization and mortality in these patients at 1-year follow up [39]. Here, at least one of the MACE considered (MI, ischemic stroke, hospitalization due to unstable angina, and urgent coronary revascularization) was documented in over a third of patients during follow up. The 5-year cumulative incidence of MACE was 35.6% (rate of 225.7 per 1,000 person-year), with an incidence of MI of 17.5% (rate of 63.7 per 1,000 person-year), and ischemic stroke of 5% (rate of 16.9 per 1,000 person-year). In this line, the ATHENA study analyzed two cohorts of patients with T2D, namely patients at high cardiovascular risk (THEMIS-like cohort; n = 56,040) and patients at high cardiovascular risk or taking P2Y12 inhibitors (CAD-T2D cohort; n = 69,790). In ATHENA, the event rates in 100 person-years (THEMIS-like vs. CAD-T2D cohorts) for the composite outcome were 16.34 (95% CI: 16.31–16.37) vs. 17.64 (17.61–17.67), for MI 5.2 (5.19–5.23) vs. 5.5 (5.49–5.52), and for ischemic stroke 5.3 (5.37–5.41) vs 6.1 (6.11–6.15) [31]. Patients in the THEMIS-like cohort and the broader CAD-T2D population had substantial cardiovascular event rates, in turn indicating these patients are at an increased cardiovascular risk [13]. However, the high rates of ischemic events (MI and ischemic stroke) in the REACH and ATHENA studies, as well as in the present study, are likely attributable to the clinical profile of the patients enrolled, with a high proportion of elderly patients with multiple comorbidities. In this regard, it is important to note that the nature of our study methodology did not allow us to provide realistic mortality data since we only had access to in-hospital mortality.

In our study, we found that the risk of MACE in T2D-THEMIS-like patients according to the multivariate model was associated with the extent and severity of atherothrombosis (MVD) and with other risk factors such as history of heart valve disease and respiratory disorders (COPD/asthma). These risk factors have previously been shown to affect outcomes in general population and diabetic patients at high risk for ischemic events, specifically in patients with T2D and CAD with no history of MI or stroke and previous PCI revascularization [40–43]. Finally, the multivariate approach also revealed that such treatments as clopidogrel and statins were associated to a lower risk for MACE after adjusting for other factors in this clinical population; further research is warranted to confirm these findings in future studies.

## Limitations

The results of the present study should be interpreted in light of the following limitations. First, our results are based on data captured directly from the unstructured, free-text narratives in patients’ EHRs. These findings are limited by the availability and accuracy of EHRs, and by the actual information that is jotted down by physicians in their routine practice. In this context, it is difficult to differentiate between “true zero” values, missing data, or unspecified information. Second, unlike classical studies or clinical trials, this was a retrospective analysis based on real-world data. The availability of an electronic record for a given patient does not guarantee that all desired variables will be present. Similarly, we captured information from patients with both single and multiple hospital visits, which may contribute to the heterogeneity of the data. Third, regarding laboratory results and other variables with substantial missing datapoints, it should be noted that physicians might not explicitly include this information as free text, but instead capture general assessments or an overall conclusion regarding the patients’ health status. Finally, the MACE events included in the analyses were limited by the information available in EHRs. In this line, all-cause and cardiovascular mortality were not reported since we only had access to in-hospital mortality.

## Conclusions

This retrospective, observational, and real-world study represents the first attempt to combine NLP and machine learning to explore the unstructured information from EHRs in such a large series of PCI-revascularized patients with T2D and CAD with no history of MI or stroke in Spain. Using a multicenter approach, we were able to collect large amounts of patients’ longitudinal information, describe the clinical profile of these patients, and establish associations between MACE and clinical variables. Our results showed substantial rates of cardiovascular events in THEMIS-PCI-like Spanish patients. Regarding current management and risk factors for cardiovascular events, we replicated previously published findings based on traditional research approaches while offering new insights and hypothesis that could be explored in clinical trials and routine clinical practice studies.

## Supporting information

S1 File (RTF)

## Abbreviations

AEMPS: Spanish Agency of Medicines and Health Products
ACE: Angiotensin-converting enzyme
AIC: Akaike information criterion
ARBs: Angiotensin II receptor blockers
ASA: Acetylsalicylic acid
CABG: Coronary artery bypass graft
CAD: Coronary artery disease
CHD: Coronary Heart Disease
CKD: Chronic Kidney Disease
COPD: Chronic obstructive pulmonary disease
CRT: Cardiac Resynchronization Therapy
CVD: Cardiovascular diseases
DPP4i: Dipeptidyl peptidase 4 inhibitors
EHRs: Electronic Health Records
GLP1: Glucagon-like peptide-1
FA: Fast Acting
ICD: Implantable Cardioverter Defibrillator
IA: Intermediate-Acting
SGLT2i: Sodium glucose co-transporter 2 inhibitors
LA: Long-Acting
LMACD: Left Main Artery Coronary Disease
T2D: Type 2 diabetes mellitus
MACE: Major adverse cardiovascular events (MI, ischemic stroke, urgent coronary revascularization, and hospitalization due to unstable angina)
MI: Myocardial infarction
MVD: Multivessel Disease Subgroup
NLP: Natural Language Processing
P: Precision
PAD: Peripheral Artery Disease
PVD: Peripheral Vascular Disease
PCI: Percutaneous coronary intervention
R: Recall
SS: Subgroup Set
TIA: Transient Ischemic Attack
