# Supplementary material for: Assessment of medical management in Coronary Type 2 Diabetic patients with previous percutaneous coronary intervention in Spain: A retrospective analysis of electronic health records using Natural Language Processing
Source: PLoS One. 2022 Feb 10;17(2):e0263277. doi: 10.1371/journal.pone.0263277 (PMC8830700; doi:10.1371/journal.pone.0263277)
Supplement: S1 File — (RTF) [file pone.0263277.s001.rtf]

Assessment of medical management in Coronary Type 2 Diabetic patients with previous percutaneous coronary intervention in Spain: A retrospective analysis of electronic health records using Natural Language Processing


SUPPLEMENTAL MATERIALS


Supplemental Methods
1.	Evaluation of EHRead®'s performance
Due to the novel methodological approach used in this study, we complemented our clinical findings with an evaluation of the performance of EHRead®. The goal of this evaluation was to verify the system's accuracy when identifying records that contain mentions of coronary disease and related clinical terms.
Our evaluation required the development of an annotated corpus known as annotation gold standard. This is a set of documents marked up with metadata tags related to the study objectives. Developing the annotation gold standard required the following phases: 
·	Text collection. The amount of data necessary to capture enough linguistic events to be able to produce consistent performance measures is an open question in NLP. To determine the size of a corpus that captures the population characteristics as closely as possible while overcoming size limitations, we used the SampLe Calculator for the Evaluation (SLiCE) tool. This calculator, developed by Savana, indicates the minimum number of annotated EHRs required to obtain the expected levels of confidence, based on the prevalence in the EHRs of the main study variable (in this case, coronary disease). The parameters used for this calculation include a confidence level of 95% (á = 5%), interval widths of 10% (percentage points) and expected values of precision (P) and recall (R). In other words, this methodology provides a robust estimation of P and R assuring that the true value is at ±5% (pp) with a confidence level of 95%. For the rest of the variables of the study, the sample size is not calculated, this being subject to the one calculated for the variable 'coronary disease'. 
·	Annotation task. To build the gold standard corpus, a set of documents was first pre-annotated using Savana's EHRead technology; these documents only included the key clinical variables described above. These documents were then corrected manually via Savana's Evaluation Tool. The overall goal of this phase is to evaluate the system's accuracy when identifying records that contain mentions of coronary disease and its related variables.
·	Annotation of the gold standard. Two designated expert physicians (hereby referred to as 'the annotators') at each hospital annotated the set of randomly selected records. In this study, a total of 1,250 records were annotated by expert physicians. Annotators followed the annotation guidelines written by Savana's medical team. Once the annotations were finalized, the Inter-Annotator Agreement (IAA) was measured using the F1-Score to ensure the consistency of the guidelines and the reliability of the annotation. The IAA is a metric that indicates the extent to which the different annotators converged in their evaluation, thus also providing information regarding the difficulty of the task. Finally, a third physician acted as judge, reviewing the annotations made by the two annotators and resolving any possible discrepancies. The resulting gold standard corpus served as a resource for the evaluation of the performance of Savana technology. 
·	Evaluation. The evaluation of the system is calculated in terms of the standard metrics of Precision (P), Recall (R), and their harmonic mean F1-Score
o	Precision = . This parameter indicates the accuracy of the system in retrieving key clinical concepts.
o	Recall =  . This parameter indicates the amount of information the system retrieves.
o	F1-Score = . This parameter gives us an overall performance indicator of information retrieval.
In all cases, tp is the number of true positives (i.e., records correctly retrieved), fn is the set of false negatives (i.e., records incorrectly not retrieved), and fp is the number of false positives (i.e., records incorrectly retrieved).
The results of EHRead's performance metrics are shown in Table S1.
Supplemental Tables

Table S1. Performance of EHRead® identifying records that contain key CAD-related variables
Variable	Recall (R)	Precision (P)	F1-Score	
Coronary disease	0.7347368421	0.885786802	0.8032220944	
T2D	0.7485294118	0.9960861057	0.8547439127	
Revascularization 	0.6299212598	0.9552238806	0.7591933571	
Arterial hypertension	0.96111665	0.9887179487	0.9747219414	
Heart failure	0.8925081433	0.9547038328	0.9225589226	
Diabetic foot	0.9928057554	0.9928057554	0.9928057554	
Peripheral artery disease	0.6984924623	0.9586206897	0.8081395349	
NSTE ACS	0.7739726027	1	0.8725868726	
STE ACD	0.9140625	0.8731343284	0.893129771	
Acute myocardial infarction	0.8582278481	0.976945245	0.9137466307	
Ictus	0.7784431138	0.9420289855	0.8524590164	
Acute ischemic stroke	0.990990991	0.990990991	0.990990991	
Coronary angiography	0.7864693446	0.9867374005	0.8752941176	
Dapagliflozine	0.9927536232	1	0.9963636364	
Enalapril	0.9433962264	0.9689922481	0.9560229446	
Acenocumarol	0.9702970297	0.9671052632	0.9686985173	
Atenolol	0.9507042254	0.9926470588	0.9712230216	
HbA1c	0.880952381	0.9830917874	0.9292237443	
LDL	0.8980392157	0.9978213508	0.9453044376	
HDL	0.9445585216	0.9956709957	0.9694415174	
TOTAL*	0.856228727	0.9730775182	0.9109212051	
*Aggregated value across all variables. NSTE ACS = Non-ST segment elevation acute coronary syndrome; STE ACS = ST segment elevation acute coronary syndrome. 	


Table S2. Laboratory values at index date. 
	N = 1,579	
Hematogram		
Hemoglobin (g/dl)		
N	961	
Mean(SD)	13.1(2.2)	
Median 	13.2	
(Q1-Q3)	(11.7-14.6)	
Missing**	618	
Platelet count (103/mm3)		
N	739	
Mean(SD)	224.4(76.5)	
Median 	213	
(Q1-Q3)	(176-263)	
Missing**	840	
Leukocyte count (103/mm3)		
N	721	
Mean(SD)	10.1(27.9)	
Median 	8.2	
(Q1-Q3)	(6.7-10.2)	
Missing**	858	
Hematocrit (%)		
N	787	
Mean(SD)	39.6(5.6)	
Median 	39.6	
(Q1-Q3)	(36.1-43.5)	
Missing**	792	
Biochemistry		
Glucose (mg/dl)		
N	976	
Mean(SD)	152.2(67.1)	
Median 	137.5	
(Q1-Q3)	(114-172.2)	
Missing**	603	
HbA1c (%)		
N	702	
Mean(SD)	7.3(1.5)	
Median 	7.1	
(Q1-Q3)	(6.4-8)	
Missing**	877	
Total cholesterol (mg/dl)		
N	640	
Mean(SD)	148.9(41.1)	
Median 	144.5	
(Q1-Q3)	(120-168.2)	
Missing**	939	
HDL (mg/dl) #		
N	690	
Mean(SD)	42.2(20.4)	
Median 	40	
(Q1-Q3)	(33-47)	
Missing**	889	
LDL (mg/dl)		
N	738	
Mean(SD)	82.4(39.8)	
Median 	77	
(Q1-Q3)	(62-96.6)	
Missing**	841	
Triglycerides (mg/dl)		
N	720	
Mean(SD)	166.8(167.4)	
Median 	134	
(Q1-Q3)	(94.8-189.2)	
Missing**	859	
Creatinine (mg/dl)		
N	927	
Mean(SD)	1.1(1.7)	
Median 	1	
(Q1-Q3)	(0.8-1.2)	
Missing**	652	
ALT (u/l)		
N	754	
Mean(SD)	31.2(39.8)	
Median 	22	
(Q1-Q3)	(16-32)	
Missing**	825	
AST (u/l)		
N	703	
Mean(SD)	29.2(47.9)	
Median 	21	
(Q1-Q3)	(17-28)	
Missing**	876	
GFR (ml/min/1.73m2)		
N	367	
Mean(SD)	62.3(21.3)	
Median 	60	
(Q1-Q3)	(45.9-78.9)	
Missing**	1212	
CRP (mg/dl)		
N	488	
Mean(SD)	23.5(45.3)	
Median 	4.9	
(Q1-Q3)	(1.4-19.7)	
Missing**	1091	
Uric Acid (mg/dl)		
N	442	
Mean(SD)	6.3(2)	
Median 	6.1	
(Q1-Q3)	(4.9-7.5)	
Missing**	1137	
*For all laboratory results, values were extracted within the 6 months before and after the Index Date; if more than one value existed for any given patient, the closest value to the Index Date was considered for analysis. **Missing data resulting from extracting laboratory results from unstructured information captured in the EHRs.


Table S3. Cumulative incidence and rate of MACE during follow up
	N=1,579	
	CI
n(%)	Rate*	
MACE (any)	563(35.66)	225.74	
MI	277(17.54)	63.75	
Ischemic stroke	80(5.07)	16.96	
Unstable angina	112(7.09)	24.12	
Urgent revasc.	288(18.24)	66.82	
*Rate represented in x1,000 person-year. For calculations, the number of identified patients in each condition was divided by the total observation time (in years) for all patients and multiplied by 1000. CI = Cumulative Incidence 

	


Table S4. Factors associated with the occurrence of MACE during follow up
	PCI/No MACE
N=1,016	PCI/MACE
N=563	Estimate*
(CI 95%*)	P value**	
Gender n(%) ‡					
 Female 	284(27.95)	151(26.82)	 1.07 (0.84, 1.36)	0.598	
 Male 	728(71.65)	411(73)	 0.94 (0.74, 1.20)	0.639	
Age (years) #					
 N 	1016	563			
 Mean(SD) 	70.5(10)	70.4(9.9)	 0.00 (-1.00, 1.00)	0.809	
 Median 	71	71			
 (Q1-Q3) 	(64-79)	(64-78)			
 Missing 	4	1			
Tobacco Use ‡					
Former smoker	488(48.03)	275(48.85)	 1.28 (0.94, 1.76)	0.115	
No/Unknown 	409(40.26)	206(36.59)	 0.86 (0.69, 1.06)	0.161	
Yes	119(11.71)	82(14.56)	 1.03 (0.84, 1.28)	0.793	
T2D: Time since first mention in EHRs #					
 N 	1016	563			
 Mean(SD) 	4.3(6.4)	5.2(7)	 0.19 (0.01, 0.51)	0.001**	
 Median 	2.1	2.6			
 (Q1-Q3) 	(0.5-4.8)	(0.8-6)			
 Missing 	0	0			
CAD: Time since first mention in EHRs #					
 N 	1016	563			
 Mean(SD) 	3.1(4.3)	3.2(4.6)	 0.21 (0.08, 0.36)	0.001**	
 Median 	1.3	1			
 (Q1-Q3) 	(0.4-4.3)	(0-4.7)			
 Missing 	0	0			
Follow-up duration #					
 N 	1016	563			
 Mean(SD) 	2.9(1.4)	3.2(1.4)	-0.02 (-0.26, 0.00)	0.043**	
 Median 	3(0.5-5)	3.2(0.5-5)			
 (Q1-Q3) 	(1.8-4.2)	(2-4.5)			
 Missing 	0	0			
Comorbidities ‡					
 Blood and lymphatic system disorders					
 Anemia	186(18.31)	104(18.47)	 1.01 (0.77, 1.33)	0.946	
 Cardiovascular disorders					
 Transient Ischemic Attack (TIA)	24(2.36)	20(3.55)	 1.52 (0.79, 2.90)	0.201	
 Arterial hypertension	894(87.99)	502(89.17)	 1.12 (0.80, 1.58)	0.512	
 Moderate/severe LV systolic dysfunction	76(7.48)	51(9.06)	 1.23 (0.83, 1.81)	0.288	
 Heart Failure	209(20.57)	152(27)	 1.43 (1.11, 1.83)	0.004**	
 Atrial Flutter	174(17.13)	96(17.05)	 0.99 (0.75, 1.32)	1.000	
 Atrial fibrillation	135(13.29)	77(13.68)	 1.03 (0.75, 1.41)	0.818	
 Heart Valve Disease	410(40.35)	249(44.23)	 1.17 (0.95, 1.45)	0.136	
 Multivessel Coronary Disease	470(46.26)	302(53.64)	 1.34 (1.09, 1.66)	0.005**	
 Peripheral Vascular Disease 	382(37.6)	238(42.27)	 1.22 (0.98, 1.51)	0.076	
  Other/Unknown	363(35.73)	232(41.21)	-	-	
  Peripheral Artery Disease (PAD)	164(16.14)	111(19.72)	 1.28 (0.97, 1.68)	0.083	
   Claudication	78(7.68)	59(10.48)	 1.41 (0.97, 2.04)	0.062	
   Foot or leg cellulitis-osteomyelitis	9(0.89)	7(1.24)	 1.41 (0.44, 4.28)	0.601	
   Other/Unknown	124(12.2)	91(16.16)	-	-	
 Angina	693(68.21)	389(69.09)	 1.04 (0.83, 1.31)	0.735	
Unstable angina	352(34.65)	212(37.66)	 1.14 (0.91, 1.42)	0.250	
Stable angina 	329(32.38)	190(33.75)	 1.06 (0.85, 1.33)	0.615	
 Eye disorders					
 Diabetic retinopathy	63(6.2)	55(9.77)	 1.64 (1.10, 2.43)	0.012**	
 Endocrine, metabolism and nutrition disorders	615(60.53)	353(62.7)	 1.10 (0.88, 1.36)	0.419	
 Hyperlipidemia	401(39.47)	237(42.1)	 1.11 (0.90, 1.38)	0.310	
 Hypoglycemia	43(4.23)	26(4.62)	 1.10 (0.64, 1.85)	0.702	
 Gout	40(3.94)	25(4.44)	 1.13 (0.65, 1.94)	0.692	
 Hyperthyroidism	17(1.67)	10(1.78)	 1.06 (0.43, 2.48)	0.843	
 Hypothyroidism	69(6.79)	35(6.22)	 0.91 (0.58, 1.41)	0.751	
 Obesity	307(30.22)	178(31.62)	    1.07 (0.85, 1.34)	0.569	
Gastrointestinal and hepatobiliary disorders					
 Chronic liver disease	19(1.87)	10(1.78)	 0.95 (0.39, 2.16)	1.000	
 Musculoskeletal and connective tissue disorders					
 Diabetic foot	7(0.69)	2(0.36)	 0.51 (0.05, 2.71)	0.504	
 Nervous system disorders					
 Diabetic neuropathy	27(2.66)	14(2.49)	 0.93 (0.45, 1.86)	1.000	
 Psychiatric disorders					
 Depression/Anxiety	155(15.26)	94(16.7)	   1.11 (0.83, 1.48)	0.471	
 Renal and urinary disorders	154(15.16)	81(14.39)			
 CKD (Chronic Kidney Disease)	154(15.16)	81(14.39)	 0.94 (0.69, 1.27)	0.712	
 Reproductive system and breast disorders					
 Erectile dysfunction	21(2.07)	14(2.49)	 1.21 (0.56, 2.51)	0.596	
 Respiratory/mediastinal disorders	238(23.43)	160(28.42)	   1.30 (1.02, 1.65)	0.030**	
 COPD/Asthma 	155(15.26)	114(20.25)	 1.41 (1.07, 1.86)	0.014**	
 Sleep apnea	130(12.8)	73(12.97)	 1.02 (0.74, 1.39)	0.938	
T2D-related medication ‡					
Oral hypoglycemic agents	1016(100)	563(100)			
Metformin	801(78.84)	448(79.57)	 1.05 (0.81, 1.36)	0.747	
iDPP4 	192(18.9)	114(20.25)	 1.09 (0.83, 1.42)	0.550	
iSGLT2 	25(2.46)	14(2.49)	 1.01 (0.48, 2.04)	1.000	
GLP1-RA 	38(3.74)	29(5.15)	 1.40 (0.82, 2.36)	0.194	
Sulfonylureas	222(21.85)	143(25.4)	 1.22 (0.95, 1.56)	0.119	
Glinides 	111(10.93)	64(11.37)	1.05 (0.74, 1.65)	0.802	
Thiazolidinediones	17(1.67)	13(2.31)	 1.39 (0.62, 3.06)	0.442	
Alpha-glucosidase	14(1.38)	2(0.36)	 0.26 (0.03, 1.12)	0.065	
Combination therapies	229(22.54)	140(24.87)	 1.14 (0.89, 1.46)	0.321	
Insulin treatment 	225(22.15)	188(33.39)	 1.76 (1.39, 2.23)	<0.001**	
FA	75(7.38)	60(10.66)	 1.50 (1.03, 2.17)	0.030**	
IA	22(2.17)	19(3.37)	 1.58 (0.80, 3.08)	0.185	
IA/LA + FA	52(5.12)	47(8.35)	 1.69 (1.10, 2.59)	0.013**	
LA	167(16.44)	138(24.51)	 1.65 (1.27, 2.14)	<0.001**	
CAD-related medication ‡					
Anticoagulant therapy	179(17.62)	110(19.54)	 1.14 (0.86, 1.49)	0.343	
Vitamin-k antagonist	123(12.11)	62(11.01)	 0.90 (0.64, 1.25)	0.568	
Warfarin 	1(0.1)	1(0.18)	  1.81 (0.02, 141.75)	1.000	
Acenocoumarol 	123(12.11)	62(11.01)	 0.90 (0.64, 1.25)	0.568	
Non-vitamin-k antagonist oral 	107(10.53)	68(12.08)	 1.17 (0.83, 1.63)	0.358	
Heparin group	68(6.69)	53(9.41)	 1.45 (0.98, 2.14)	0.060	
Direct thrombin inhibitors	25(2.46)	9(1.6)	 0.64 (0.26, 1.44)	0.284	
Direct factor Xa inhibitors	13(1.28)	10(1.78)	 1.39 (0.54, 3.47)	0.511	
Fondaparinux	9(0.89)	5(0.89)	 1.00 (0.26, 3.35)	1.000	
Antiplatelet agents	966(95.08)	511(90.76)	 0.51 (0.33, 0.78)	0.001**	
ASA	883(86.91)	473(84.01)	 0.79 (0.59, 1.07)	0.114	
Clopidogrel	595(58.56)	309(54.88)	 0.86 (0.70, 1.07)	0.167	
Prasugrel	39(3.84)	23(4.09)	 1.07 (0.60, 1.85)	0.789	
Ticagrelor	65(6.4)	23(4.09)	 0.62 (0.37, 1.03)	0.066	
Other	120(11.81)	69(12.26)	 1.04 (0.75, 1.44)	0.808	
DAPT	526(51.77)	264(46.89)	 0.82 (0.67, 1.02)	0.066	
Clopidogrel + ASA	433(42.62)	221(39.25)	 0.87 (0.70, 1.08)	0.201	
Prasugrel + ASA	34(3.35)	16(2.84)	 0.84 (0.43, 1.59)	0.654	
Ticagrelor + ASA	46(4.53)	18(3.2)	 0.70 (0.38, 1.24)	0.231	
Other cardiovascular therapy	1001(98.52)	550(97.69)	 0.63 (0.28, 1.46)	0.237	
Beta Blockers	783(77.07)	428(76.02)	 0.94 (0.74, 1.21)	0.664	
ACE inhibitors or ARB	876(86.22)	486(86.32)	 1.01 (0.74, 1.38)	1.000	
Calcium channel Blockers	474(46.65)	276(49.02)	 1.10 (0.89, 1.36)	0.372	
Nitrates	520(51.18)	306(54.35)	 1.14 (0.92, 1.40)	0.247	
Ivabradine	80(7.87)	53(9.41)	 1.22 (0.83, 1.77)	0.299	
Ranolazines	87(8.56)	63(11.19)	 1.35 (0.94, 1.92)	0.090	
Diuretics	423(41.63)	275(48.85)	 1.34 (1.08, 1.66)	0.006**	
Lipid lowering drugs	953(93.8)	512(90.94)	 0.66 (0.44, 1.00)	0.042**	
Statins	937(92.22)	492(87.39)	 0.58 (0.41, 0.83)	0.002**	
Other lipid lowering drugs	228(22.44)	142(25.22)	 1.17 (0.91, 1.49)	0.215	
*Estimates represent odds ratios (OR) for Fisher tests (‡) and location difference for Wilcox tests (#). CI = Confidence Interval. **Significant differences between MACE and no-MACE were considered when p < 0.05 in two-tailed tests. DAPT refers to ASA plus other anti-platelet drug. ACE = angiotensin-converting enzyme inhibitors; ARB = angiotensin II receptor blockers; ASA = acetylsalicylic acid; DAPT=dual antiplatelet therapy; DPP4i = dipeptidyl peptidase 4 inhibitors; SGLT2i = sodium-glucose cotransporter 2 inhibitors ; GLP1 = Glucagon-like peptide-1; FA = Fast acting; IA = Intermediate acting; LA = Long acting.	
